# Splenic switch-off as a predictor for coronary adenosine response: validation against 13N-ammonia during co-injection myocardial perfusion imaging on a hybrid PET/CMR scanner

**DOI:** 10.1186/s12968-020-00696-y

**Published:** 2021-01-07

**Authors:** Dimitri Patriki, Elia von Felten, Adam Bakula, Andreas A. Giannopoulos, Christel H. Kamani, Moritz Schwyzer, Michael Messerli, Dominik C. Benz, Catherine Gebhard, Christoph Gräni, Aju P. Pazhenkottil, Philipp A. Kaufmann, Tobias A. Fuchs, Ronny R. Buechel

**Affiliations:** Department of Nuclear Medicine, Cardiac Imaging, University Hospital Zurich and University Zurich, Ramistrasse 100, 8091 Zurich, Switzerland

**Keywords:** Splenic switch-off, Cardiovascular magnetic resonance imaging, Myocardial perfusion imaging

## Abstract

**Background:**

Inadequate coronary adenosine response is a potential cause for false negative ischemia testing. Recently, the splenic switch-off (SSO) sign has been identified as a promising tool to ascertain the efficacy of adenosine during vasodilator stress cardiovascular magnetic resonance imaging (CMR). We assessed the value of SSO to predict adenosine response, defined as an increase in myocardial blood flow (MBF) during quantitative stress myocardial perfusion 13 N-ammonia positron emission tomography (PET).

**Methods:**

We prospectively enrolled 64 patients who underwent simultaneous CMR and PET myocardial perfusion imaging on a hybrid PET/CMR scanner with co-injection of gadolinium based contrast agent (GBCA) and 13N-ammonia during rest and adenosine-induced stress. A myocardial flow reserve (MFR) of  > 1.5 or ischemia as assessed by PET were defined as markers for adequate coronary adenosine response. The presence or absence of SSO was visually assessed. The stress-to-rest intensity ratio (SIR) was calculated as the ratio of stress over rest peak signal intensity for splenic tissue. Additionally, the spleen-to-myocardium ratio, defined as the relative change of spleen to myocardial signal, was calculated for stress (SMR_stress_) and rest.

**Results:**

Sixty-one (95%) patients were coronary adenosine responders, but SSO was absent in 18 (28%) patients. SIR and SMR_stress_ were significantly lower in patients with SSO (SIR: 0.56 ± 0.13 vs. 0.93 ± 0.23; p < 0.001 and SMR_stress_: 1.09 ± 0.47 vs. 1.68 ± 0.62; p < 0.001). Mean hyperemic and rest MBF were 2.12 ± 0.68 ml/min/g and 0.78 ± 0.26 ml/min/g, respectively. MFR was significantly higher in patients with vs. patients without presence of SSO (3.07 ± 1.03 vs. 2.48 ± 0.96; p = 0.038), but there was only a weak inverse correlation between SMR_stress_ and MFR (R = -0.378; p = 0.02) as well as between SIR and MFR (R = -0.356; p = 0.004).

**Conclusions:**

The presence of SSO implies adequate coronary adenosine-induced MBF response. Its absence, however, is not a reliable indicator for failed adenosine-induced coronary vasodilatation.

## Background

Due to its rapid onset of action, its short half-life and the good tolerability and safety profile, adenosine is a widely used vasodilator for ischemia testing in myocardial perfusion imaging (MPI) using single photon emission computed tomography (SPECT), positron emission tomography (PET), and cardiovascular magnetic resonance imaging (CMR). However, induction of maximal coronary vasodilation is a crucial prerequisite for obtaining high diagnostic accuracy concerning the detection of obstructive coronary artery disease (CAD) [1–4]. Whether this condition is achieved remains difficult to assess in clinical routine because commonly used markers such as the hemodynamic response to adenosine (i.e., a decrease in systolic blood pressure and/or increase in heart rate) are unreliable as they are prone to procedure-related confounders such as anxiety or adenosine-induced side effects [5, 6]. Recently, the splenic switch-off (SSO) sign has been proposed as a more objective and direct marker of adequate adenosine response during stress CMR [7]. SSO has been defined as a visible decrease in splenic signal intensity during adenosine stress as compared to rest, presumably due to reduced splenic blood flow which may be mediated through reactive sympathetic vasoconstriction after adenosine-induced hypotension [8].

Contrary to modalities relying on the detection of relative regional differences in myocardial perfusion, such as CMR and SPECT, PET MPI allows for absolute quantification of myocardial blood flow (MBF) and calculation of myocardial flow reserve (MFR) [9]. Adenosine-induced coronary vasodilation has been shown to increase MBF during PET MPI up to fourfold over resting baseline [10]. Hence, PET-derived MBF quantification is a reliable standard of truth for assessing adequate coronary adenosine response. Furthermore, the introduction of novel PET/CMR devices, incorporating both modalities in a single hybrid scanner, offers the unique possibility of simultaneous assessment and cross-validation of both modalities during co-injection of gadolinium based contrast agents (GBCA) and 13N-ammonia [11]. Most importantly, the simultaneity of PET and CMR data acquisition during the same adenosine stimulus ensures an identical physiological state of the coronary and extracardiac vasculature. We sought to test the hypothesis that SSO constitutes a reliable predictor of adequate coronary adenosine response. MFR served as the standard of truth and was assessed on a PET/CMR device with co-injection of 13N-ammonia and GBCA during adenosine stress.

## Methods

### Study design and population

Data of this prospective single-center study were derived from ongoing PET/CMR projects. We assessed patients who underwent cardiac PET/CMR for evaluation of CAD or cardiomyopathy. Written informed consent was obtained from all participants, and the study protocol was approved by the local ethics committee (KEK-ZH-Nr. 2014-0187 and BASEC-Nr. 2018-00170). Patients aged ≥ 18 years without any contraindications against CMR (e.g., implanted cardiac devices, claustrophobia, known GBCA allergy, severe renal impairment), adenosine (e.g., asthma, atrioventricular block), or PET (e.g., pregnancy or breastfeeding) were included. This study was partially funded by the Swiss National Science Foundation (SNSF Project Nr. 175640).

### Hybrid PET/CMR perfusion imaging

CMR and PET datasets were acquired using a hybrid PET/CMR device incorporating a 3 T CMR and a latest-generation PET scanner with time-of-flight (TOF) (Signa PET/MR, GE Healthcare, Waukesha, Wisconsin, USA). All patients were asked to refrain from caffeine intake for at least 12 h before the examination. The stress protocol consisted of 6 min of adenosine infusion with a weight-adapted dose of adenosine (140 μg/kg/min). A body mass index adapted dose of 13N-ammonia (i.e., 200–600 megabecquerels (MBq) and a weight-adapted dose of GBCA (Gadovist, Bayer AG, Berlin, Germany) (0.1 mmol/kg) was simultaneously injected 3 min into adenosine stress. Dynamic PET data acquisition consisted of 21 frames (i.e., 9 × 10-s, 6 × 15-s, 3 × 20-s, 2 × 30-s, and 1 × 120-s), followed by an electrocardiogram (ECG)-gated static acquisition over 10 min. Resting perfusion imaging was performed using an identical acquisition protocol after a minimum of 15 min following the stress acquisition according to Society for Cardiovascular Magnetic Resonance (SCMR) guidelines [12]. All PET data were acquired in 3D mode and reconstructed using TOF reconstruction with VUE Point FX (2 iterations and 16 subsets) and 5-mm Hanning filter. Standard DIXON-based maps were used for attenuation correction [13]. Summed semi-quantitative myocardial PET tracer uptake and quantitative MBF was obtained from stress and rest scans and analyzed using QPET (Version 2015, Cedars-Sinai Medical Center, Los Angeles, California, USA). Datasets were examined in consensus by two experienced readers regarding the presence of ischemia and/or scar.

CMR stress and rest perfusion scans were acquired using three left ventricular short-axis slices per cardiac cycle (basal, mid, and apical levels) each of 10 mm slice thickness and ECG-gated breath-hold protocol. A T1 weighted fast gradient echo sequence with short TR and TE (TR 3.3 ms TE 1.2 ms, flip angle 20°) was used for stress and rest perfusion with a typical acquired voxel size of 2.9 × 2.9 mm^2^ and typical matrix size of 128 × 128 (frequency × phase). A 90-degree non-selective saturation preparation pulse was applied prior to every acquired slice with a saturation delay time of 100 ms. A parallel imaging acceleration factor of 2 was used to reduce slice acquisition time. Heart rate (HR) and blood pressure were monitored during the stress and rest scans. A HR increase ≥ 10 beats per minute (bpm) was regarded as a positive HR response to adenosine [14]. However, as HR response has been shown to be influenced by several parameters like age and sex [15], we used MFR as assessed by PET to define true coronary adenosine response. Taking into account a reported day-to-day intra-subject variability of MFR of up to 20% [16], we defined an MFR of > 1.5 or the presence of ischemia on PET as markers for adequate coronary adenosine response.

### Visual and quantitative assessment of SSO

Presence of SSO was defined as a visually perceivable lower splenic enhancement on stress than on rest first-pass CMR images, as previously reported [7]. Visual analysis was performed independently by two readers in a blinded fashion. In cases of disagreement between the readers, the decision was made by consensus. Inter-rater variability was calculated using Cohen’s Kappa (κ). Greyscale values were normalized, and identical thresholds were used for stress and rest images for better comparability using a commercially available software package (Circle 42, Version 5.6.4, Circle Cardiovascular Imaging, Calgary, Canada).

**Fig. 1:**
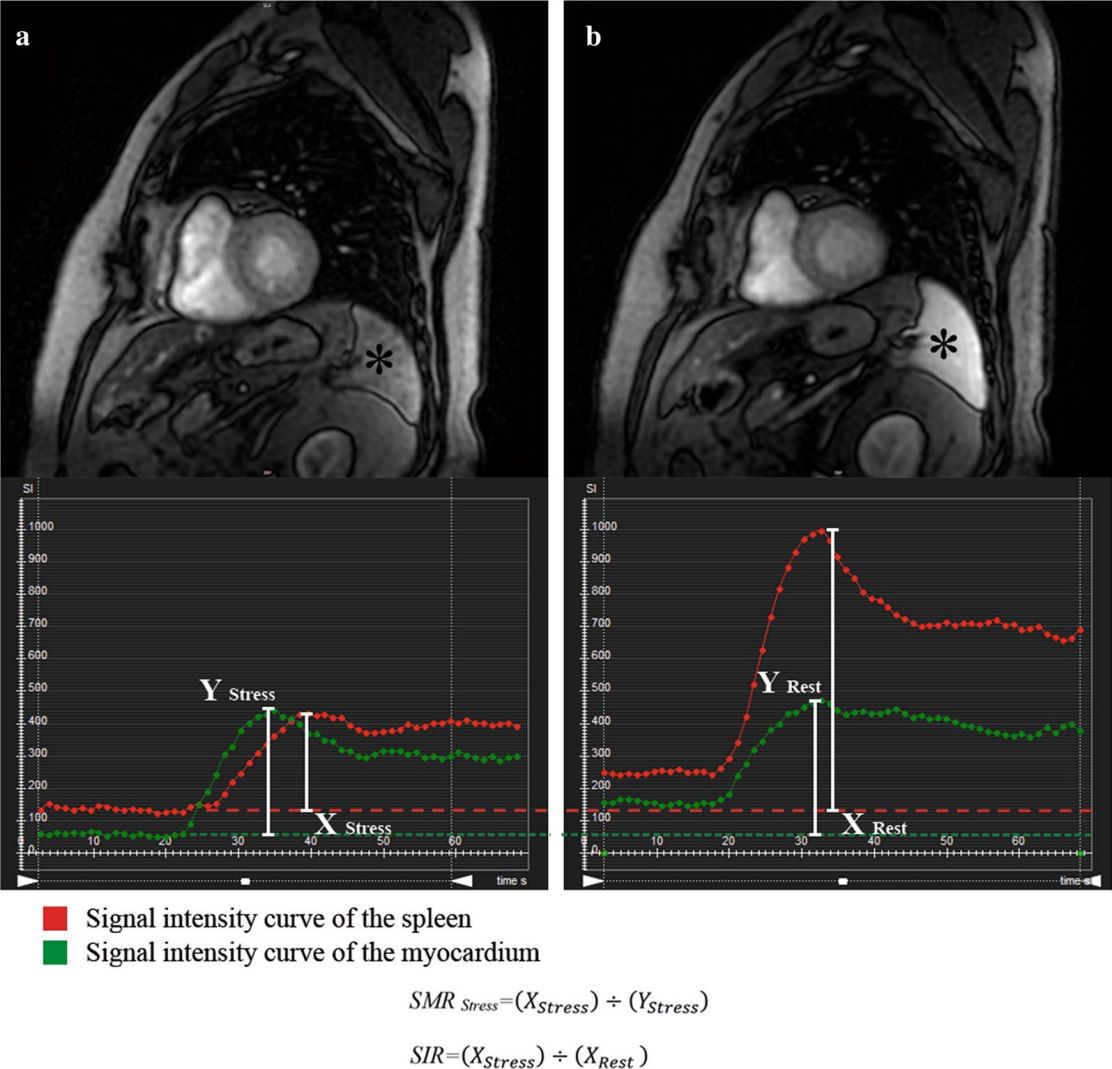
Example of presence of splenic switch-off sign (SSO) (*) with signal intensity curves. **a** Stress perfusion, **b** rest perfusion. *SMR*  spleen-myocardium intensity ratio, *SIR* stress-to-rest intensity ratio for spleen

Myocardial signal intensity curves during first-pass perfusion were derived from CMR. In addition, regions of interest were drawn in the spleen. Corrected peak signal intensity of the spleen was calculated for stress and rest by subtracting baseline intensity (pre-contrast) from peak intensity after first-pass perfusion. Stress-to-rest intensity ratio (SIR) was calculated as the ratio between corrected peak signal intensity of stress and rest images for splenic tissue as previously reported (Fig. 1) [14]. Finally, spleen-to-myocardium intensity ratios for stress (SMR_stress_) were calculated, defined as the ratio of increase of spleen activity to myocardial intensity during stress and rest, respectively, to ensure standardized evaluation (Fig. 1).

### Statistical analysis

Descriptive statistics were used to examine clinical data. Values are presented as averages ± standard deviations or as total numbers and as percentages, where applicable. Patients were stratified into groups according to the presence or absence of SSO and according to a positive or negative HR response (i.e., ≥ or < 10 bpm increase upon adenosine stress). Comparison between the groups was performed using a Chi2-test in nominal or students’ t-test and Mann–Whitney-test in normally distributed and non-parametric variables, respectively. Correlation analysis between SIR and MFR, SMR and MFR, as well as between the HR increase from rest to stress and MFR was performed using Pearson analysis. A cut-off value for SIR and SMR for predicting the presence of SSO was calculated using receiver operating characteristic (ROC) analysis. Statistical analysis was performed using SPSS (version 22, Statistical Package for the Social Sciences, International Business Machines, Inc., Armonk, New York, USA).

## Results

### Baseline characteristics

A total of 67 patients were included in our study. The spleen was visible within the CMR field-of-view in 64 (96%) patients. The remaining 3 (4%) patients were excluded from further investigations. Baseline characteristics and PET findings are given in Table 1.

**Table 1:** Baseline characteristics and comparison of PET results of patients with positive vs negative splenic switch off

	All patients n = 64	SSO present n = 46 (72%)	SSO absent n = 18 (28%)	p-Value
*Baseline characteristics*				
Mean age (years)	51 ± 20	51.4 ± 18	49.2 ±24	0.69
Male gender	52 (81)	37 (80)	15 (83)	0.30
Weight (kg)	80 ± 11	81 ± 10	75 ± 12	0.047
Height (cm)	175 ± 8	175 ± 8	176 (± 8	0.65
Body mass index(kg/m2)	26 ± 3	27 ± 3	24 ± 3	0.006
*Risk factors*				
Diabetes mellitus	5 (7.8)	5 (11)	0 (0)	0.145
Dyslipidaemia	27 (42)	21 (46)	6 (34)	0.37
Hypertension	25 (39)	20 (43)	5 (28)	0.247
Family history	16 (25)	12 (26)	4 (23)	0.748
Smoking	21 (33)	17 (37)	4 (23)	0.259
*Medication*				
Aspirin	24 (38)	17 (37)	7(39)	0.886
Betablocker	18 (28)	15 (33)	3 (17)	0.202
ACE inhibitor	24 (38)	18 (39)	6 (34)	0.667
Statin	27 (42)	19 (41)	8 (45)	0.819
*Medical history*				
Myocardial infarction	10 (16)	7 (15)	3 (17)	0.886
Stenting	18 (28)	13 (28)	5 (28)	0.969
CABG	7 (11)	4 (9)	3 (170)	0.358
*13N-ammonia PET*				
Ischemia	15 (23)	9 (20)	6 (34)	0.242
Scar	33 (52)	22 (48)	11 (61)	0.1
Stress MBF (ml/min/g)	2.12 ± 0.68	2.2 ± 0.66	1.93 ± 0.72	0.23
Rest MBF (ml/min/g)	0.78 ± 0.26	0.77 ± 0.27	0.81 ± 0.24	0.65
MFR	2.91 (± 1)	3.07 ± 1.03	2.48 ± 0.96	0.038

Values given are average ± standard deviations or absolute numbers and percentages (in brackets)

*ACE*  angiotensin converting enzyme, *CABG*  coronary artery bypass graft, *CAD*  coronary artery disease, *MBF*  myocardial blood flow, *MFR*  myocardial flow reserve. *SSO*  splenic switch-off

### 13N-ammonia PET myocardial perfusion imaging

The mean injected activity of 13N-ammonia during PET was 297 ± 98 MBq and 463 ± 103 MBq for stress and rest imaging, respectively. Mean hyperemic and rest MBF were 2.1 ± 0.7 ml/min/g and 0.8 ± 0.3 ml/min/g, respectively. Mean MFR was 2.9 ± 1.0. Three (5%) patients with an MFR ≤ 1.5 and without any signs of ischemia neither in PET nor CMR were classified as adenosine non-responders. Three (5%) additional patients with severe coronary artery disease had an MFR ≤ 1.5 but presented with clear signs of ischemia in 13 N-ammonia PET and CMR and were therefore classified as adenosine responders.

### SSO and quantitative assessment of splenic perfusion

SSO was present in 46 (72%) patients. Inter-rater reliability was excellent (κ = 0.906; p < 0.001). Among the three adenosine non-responders, two patients did not present with SSO, but one patient showed SSO. MFR differed significantly in patients with vs. patients without presence of SSO (3.1 ± 1.0 vs. 2.5 ± 1.0; p = 0.038). Splenic stress-to-rest SIR was significantly lower in patients with vs. patients without presence of SSO (SIR: 0.56 ± 0.13 vs. 0.93 ± 0.23; p < 0.001).

ROC analysis yielded an optimal cut-off value of SIR for predicting presence of SSO of 0.71 (sensitivity: 94%; specificity: 94%; AUC = 0.947). SMR_stress_ was significantly lower in patients with vs. patients without presence of SSO (1.1 ± 0.5 vs. 1.7 ± 0.6; p < 0.001). ROC analysis yielded an optimal cut-off value of SMR_stress_ for predicting presence of SSO of 1.53 (sensitivity: 61%; specificity: 85%; AUC = 0.76).

A weak correlation was found between MFR and SIR (R = -0.356; p = 0.004) as well as between MFR and SMR_stress_ (R = -0.378; p = 0.02). By contrast, there was no correlation between SMR_stresst_ or SIR and the total increase of HR during adenosine (SMR_stress_: R = -0.115; p = 0.37, SIR: R = -0.143; p = 0.26).

### HR response

HR increased significantly from rest to stress (62 ± 10 vs. 74 ± 19 bpm, p < 0.001), and 29 (45%) patients had a positive HR increase of ≥ 10 bpm. There was a trend towards higher MFR in patients with vs. patients without a positive HR response (3.2 vs 2.7 ml/min/g; p = 0.053) but there was no difference in the proportion of patients with and without a positive HR response among patients with or without SSO (p = 0.23).

## Discussion

To the best of our knowledge, this is the first study investigating the performance of SSO to predict coronary adenosine response on a hybrid PET/CMR device with co-injection of GBCA and 13N-ammonia using PET-derived MFR as the standard of truth. While we found that 95% of the patients were coronary adenosine responders, SSO was absent in 28% of the patients. Furthermore, there was only a weak correlation between SMR and MFR as well as between SIR and MFR. Hence, from our results, we conclude that the absence of SSO should not necessarily lead to the conclusion that a patient failed to respond to adenosine. By contrast, however, the presence of SSO in case of normal stress perfusion CMR is a strong indicator for a true negative finding.

Our findings are in line with the results of Kuijpers et al. who demonstrated that SSO failed to predict adenosine non-responders after caffeine intake [17]. By contrast, Manisty et al. assessed a sub-population of the CE-MARC study, comparing 35 false negative CMR scans (defined as normal CMR but significant coronary lesion in quantitative coronary angiography) with 65 true negative CMR scans in a blinded fashion [7, 18]. They found that the absence of SSO was almost four times more likely in patients with a false negative CMR than in patients with a true negative finding. Hence, the authors concluded that absence of SSO may be a predictor of inadequate coronary response to adenosine infusion [7]. However, one limitation of this study was the highly ambiguous standard of truth consisting solely of the hemodynamic response. By contrast, by performing co-injection on a PET/MR device, the current study uses a solid standard of reference in the form of PET-derived MFR.

In our study, SSO was present in 72% of the patients, a proportion which is slightly lower than previously described by Manisty et al. (90%) [7] and Hosking et al. (89%) [14]. However, our calculated thresholds of 0.71 for SIR for predicting visually perceivable SSO was higher than in the study by Hosking et al., which reported a threshold of 0.4 [14]. This more sensitive approach should have led to an even higher presence of SSO. Of note, however, both Manisty et al. [7] and Hosking et al. [14] increased the injected dose of adenosine in patients who failed to show hemodynamic changes during stress. By contrast, in the present study, we infused a fixed dose of 140 ug/kg/min. The literature on the adequate dose of adenosine reveals conflicting results with some studies raising the question whether adenosine infusion at a standard rate of 140 ul/kg/min induces maximal coronary vasodilation in all patients [19, 20]. Other studies have demonstrated that increasing the dose of adenosine beyond 140 ug/kg/min does not result in further coronary vasodilation [21, 22], but only induces a more aggravated peripheral hemodynamic response [20], potentially yielding a higher rate of SSO but without a positive effect on the coronary arteries. Hemodynamic response, as well as SSO are peripheral side effects induced by A2B receptors due to aortic hypotension with consequent reflex tachycardia and vasoconstriction of vascular beds in order to maintain mean arterial pressure. By contrast, coronary vasodilatation is induced through stimulation of A2A receptors [2]. In view of the different receptors, however, it may seem inadequate to draw conclusions as to the extent of coronary vasodilation through extrapolation of any observed peripheral effects. Moreover, accurate assessment of the splenic perfusion itself is difficult and may be hampered due to the spleen’s physiologically inhomogeneous perfusion of the red and white pulp. Hence, areas of lower and higher perfusion are simultaneously assessed by CT, ultrasound, and CMR during the arterial phase of perfusion [23, 24]. Finally, spleen visualization and analysis during myocardial perfusion imaging are currently not standardized. In the present study, one patient without SSO was classified as an adenosine responder as per the standard of reference. Of note, however, the SIR in this patient was 0.68 which lies slightly below the calculated SIR-threshold (i.e., 0.71) necessary to allow for visual recognition of SSO. This finding underlines the potential shortcomings and inherent uncertainties of a visual assessment of SSO. However, the future clinical value of SSO should be put into perspective as the advent of vasodilators such as regadenoson, which are more specific to A2A receptors and may confer a better safety profile than adenosine, may gradually reduce the clinical value of the latter in the future.

The present study extends our knowledge and understanding of the SSO sign. While our results question its value as a reliable marker of inadequate adenosine response, future prospective studies—ideally placebo- or caffeine-controlled—are also needed. It is noteworthy and important, however, that our results on the other hand also carry the reassuring message that the presence of SSO is a strong marker for adequate coronary adenosine response, therefore increasing a readers’ confidence in case of a normal CMR myocardial perfusion scan.

## Limitations

In the present study, we used a binary system to classify patients into responders vs. non-responders. This may be seen as an over-simplification in light of some studies suggesting a more graduated adenosine response, depending on receptor density and responsiveness of adenosine receptors [25].

## Conclusion

The presence of SSO implies adequate coronary adenosine-induced MBF response. Its absence, however, is not a reliable indicator for failed adenosine-induced coronary vasodilatation.

## Data Availability

The datasets used and/or analyzed during the current study are available from the corresponding author on reasonable request.

## Abbreviations

CAD: Coronary artery disease
ECG: Electrocardiogram
HR: Heart rate
GBCA: Gadolinium based contrast agent
MBF: Myocardial blood flow
MBq: Megabecquerels
MFR: Myocardial flow reserve
MPI: Myocardial perfusion imaging
PET: Positron emission tomography
ROC: Receiver operating characteristic
SIR: Stress-to-rest intensity ratio
SMR: Spleen-to-myocardium ratio
SSO: Splenic switch off
TOF: Time-of-flight
